# Sleep Quality and Quantity in Caregivers of Children with Type 1 Diabetes Using Closed-Loop Insulin Delivery or a Sensor-Augmented Pump

**DOI:** 10.1155/2023/7937007

**Published:** 2023-06-13

**Authors:** Juan J. Madrid-Valero, Julia Ware, Janet M. Allen, Charlotte K. Boughton, Sara Hartnell, Malgorzata E. Wilinska, Ajay Thankamony, Carine de Beaufort, Ulrike Schierloh, Fiona M. Campbell, Judy Sibayan, Laura E. Bocchino, Craig Kollman, Roman Hovorka, Alice M. Gregory, KidsAP Consortium

**Affiliations:** ^1^Department of Health Psychology, Faculty of Health Sciences, University of Alicante, San Vicente del Raspeig, Spain; ^2^Wellcome-MRC Institute of Metabolic Science, University of Cambridge, Cambridge, UK; ^3^Department of Paediatrics, University of Cambridge, Cambridge, UK; ^4^Wolfson Diabetes and Endocrine Clinic, Cambridge University Hospitals NHS Foundation Trust, Cambridge, UK; ^5^DECCP, Clinique Pédiatrique, Centre Hospitalier de Luxembourg, Luxembourg City, Luxembourg; ^6^Department of Pediatric Endocrinology, University Hospital Brussels, Brussels, Belgium; ^7^Department of Pediatric Diabetes, Leeds Children's Hospital, Leeds, UK; ^8^Jaeb Center for Health Research, Tampa, Florida, USA; ^9^Department of Psychology, Goldsmiths, University of London, London, UK; ^10^University of Cambridge, Cambridge, UK

## Abstract

**Introduction:**

Parents of children living with type 1 diabetes (T1D) often report short and/or poor quality sleep. The development of closed-loop systems promises to transform the management of T1D. This study compared sleep quality and quantity in caregivers of children using a closed-loop system (CL) or sensor-augmented pump (SAP) therapy.

**Method:**

Data from sleep diaries, accelerometers, and questionnaires were provided by forty parents (classified as caregiver 1 (main analyses) or 2 (supplementary analyses) based on their contribution towards treatment management) of 21 very young children aged 1 to 7 years living with T1D (mean age: 4.7 (SD = 1.7)). Assessments were made at a single post-randomisation time point when the child was completing either the 16-week CL arm (*n* = 10) or the 16-week SAP arm (*n* = 11) of the main study.

**Results:**

Overall, there was a mixed pattern of results and group differences were not statistically significant at the *p* < 0.05 level. However, when we consider the direction of results and results from caregiver 1, sleep diary data showed that parents of the CL (as compared to the SAP) group reported a shorter sleep duration but better sleep quality, fewer awakenings, and less wake after sleep onset (WASO). Actiwatch data showed that caregiver 1 of the CL (as compared to the SAP) group had a shorter sleep latency; greater sleep efficiency; and less wake after sleep onset. Results from the Pittsburgh Sleep Quality Index also showed better sleep quality for caregiver 1 of the CL group as compared to the SAP group.

**Conclusions:**

Results from this study suggest that sleep quality and quantity in parents of children using CL were not significantly different to those using SAP. Considering effect sizes and the direction of the non-significant results, CL treatment could be associated with better sleep quality in the primary caregiver. However, further research is needed to confirm these findings. This trial is registered with NCT05158816.

## 1. Introduction

Evolutionary theories emphasise the need to be safe before falling asleep [1]. It is dangerous to lose vigilance when it is not safe to do so. Type 1 diabetes (T1D) [2] is a chronic condition where the pancreas stops producing insulin and can in itself (or as a result of treatment) result in high or low glucose levels, both of which can constitute a medical emergency. It is therefore perhaps unsurprising that parents of children living with T1D report missing out on sleep [3] or experiencing sleep of poor quality [4]. Children living with T1D themselves may also miss out on sleep or be reported to have poor quality sleep [5–7].

Factors contributing to parents missing out on sleep or experiencing sleep of poor quality are plentiful but include parental anxiety as well as practical issues such as needing to be up during the night to treat high or low glucose levels (hyperglycaemia or hypoglycaemia) [4]. Other hypothesised factors could include catching up on tasks which have not been completed during the day because of the time commitment involved with diabetes management (whether carbohydrate counting, requesting new prescriptions, changing insulin pump cannulas and cartridges, or monitoring children's activities and glucose levels). Indeed, parents have described how caring for a child living with T1D can monopolise life [8].

Given the risk of developing complications associated with T1D, including kidney disease, retinopathy, and macrovascular complications [9], a focus on parental sleep could initially appear as a relatively minor concern. However, considering sleep is crucially important—as missing out on sleep or experiencing sleep of poor quality is associated with physical health variables including cardiovascular outcomes [10] as well as a plethora of mental health variables [11], in addition to well-being [12] and occupational performance and absenteeism [13]. Moreover, when caregivers of children living with T1D miss out on sleep or experience sleep of poor quality, their ability to engage with diabetes management may be compromised [14].

Available therapies for T1D are improving all the time [9]. Recently, closed-loop (“artificial pancreas”) systems have been developed, which use both a continuous glucose monitor and an insulin pump. A computer-based algorithm increases or decreases insulin automatically depending on real-time glucose levels, with the aim of optimising glucose levels (and reducing both hyperglycaemia and hypoglycaemia) and decreasing burden. These systems have proved transformative in the care of T1D, yielding greater time with glucose in the healthy range across all paediatric age groups [15]. Indeed, the results from the full study on which the current paper is based showed that the hybrid closed-loop system improves glucose levels and quality of life in very young children, compared to the best available current therapy [16, 17].

The National Sleep Foundation recommends between 7 and 9 hours of sleep in most adults [18], and it has also been suggested that sleep efficiency <85%, sleep latency >15–30+ minutes, and more than 1 awakening at night are signs of poor sleep quality [19]. The specific management of T1D is likely to have an impact on these sleep parameters. On the one hand, introducing technology into the bedroom is against standard sleep hygiene recommendations and technology use prior to sleep can negatively impact sleep [20]. Indeed glucose warning alarms and blue light associated with mobile phones receiving information can negatively impact sleep—and one study reported poorer sleep quality in children using continuous glucose monitoring (CGM) and insulin pumps as compared to those managing their T1D in other ways [21]. On the other hand, the advantages brought by closed-loop systems including greater time with glucose levels in the healthy range and reductions in night-time hypoglycaemia and hyperglycaemia could reduce the factors impairing sleep in parents (such as the need to intervene in glucose management and helping to reduce anxiety). Indeed, it has been speculated that closed-loop systems could benefit sleep quality and quantity in both those living with T1D and their caregivers [22], although research addressing this question has provided mixed results. For example, a study of adolescents using the Medtronic 670G hybrid closed loop found that there was no difference in objective measures of sleep quantity and quality for either adolescents or their parents post-initiation of closed-loop treatment as compared to before [23]. However, parents reported an improvement in their own sleep quality after initiation as compared to before [23]. In a further study, parents of children living with T1D aged 6–13 years who were poor sleepers reported their own sleep quality to be better after using a closed-loop system (Tandem Control IQ) than beforehand [24]. Different results in this domain underscore the need to research this question further, in participants of different ages, using different closed-loop systems and using different methods to measure sleep [25].

Based on the importance of adequate sleep quality and quantity for caregivers of those living with T1D and the impact that specific types of T1D management can have on different aspects of sleep, this substudy of the KidsAP02 study looked to examine sleep quality and quantity (at a single point post-randomisation) in parents of very young children living with T1D, who were using either a closed-loop system (CL) or a sensor-augmented pump (SAP). Overall sleep quality in the children was also measured in this study to assess further possible group differences.

## 2. Methods

### 2.1. Sample

This study compared sleep quality and quantity in caregivers of a subset of children in the KidsAP02 study [26] using CL or SAP therapy. The main KidsAP02 study had a randomised two-period crossover design with two 16-week treatment periods, where children were allocated to 16 weeks of CL therapy followed by 16 weeks of SAP therapy, or vice versa in random order. Details of the design of the main study have been described previously, and detailed information about the representativeness of the full sample has been presented elsewhere [17, 26]. For the sleep substudy, participants were recruited from two UK centres (Addenbrooke's Hospital, Cambridge University Hospital NHS Foundation Trust, Cambridge, and Leeds Teaching Hospitals NHS Trust, Leeds) and one centre in Luxembourg (DECCP, Centre Hospitalier de Luxembourg, Grand Duché de Luxembourg) and comprised 40 parents of 21 children living with T1D (mean age: 4.7 (SD = 1.7; range: 2–7)) (Table 1). Parents classified themselves as caregiver 1 or 2, depending on their main role in managing their child's T1D. In this specific study, parent's sleep quality and quantity and children's overall sleep quantity reported by parents were assessed at a single post-randomisation time point when children were completing either SAP arm (*n* = 11) or CL arm (*n* = 10) of the main study (12–14 weeks after the start of the treatment; see Fuchs and Hovorka [15] for full details). This resulted in 21 participants classified as caregiver 1 (SAP = 11 and CL = 10) and 19 participants classified as caregiver 2 (SAP = 9 and CL = 10). The main body of this paper focuses on data from the primary caregiver (results for caregiver 2 are reported in a supplementary file). There were no significant differences in child age between the groups (SAP = 4.1 vs. CL = 5.0 years; *p* = 0.24).

This project was approved by the Cambridge East Research Ethics Committee (UK) and Comité National d'Ethique de Recherche (Luxembourg) and by regulatory authorities in the UK (Medicines and Healthcare products Regulatory Agency) and in Luxembourg (Ministry of Health). The study, including the sleep substudy, is registered with clinicaltrials.gov (NCT03784027).

### 2.2. Measures

#### 2.2.1. Sleep Diary

Self-reported sleep quality and quantity were measured using a sleep diary that included the following questions: (a) enter the weekday; (b) at what time did you go to bed last night?; (c) after settling down, how long did it take you to fall asleep?; (d) after falling asleep, about how many times did you wake up in the night?; (e) after falling asleep, for how long were you awake during the night in total?; (f) at what time did you finally wake up?; (g) at what time did you get up?; (h) how would you rate the quality of your sleep last night? (with five response options from very poor (1) to very good (5)); and (i) times you took off the actiwatch. Participants were asked to complete these questions during seven days (and mean scores are reported). All participants provided information for sleep diaries which was included in the full analysis.

#### 2.2.2. Actiwatch Data

Actigraphy (Philips Respironics, Bend, Oregon, USA) was used to obtain objective sleep data. Participants wore this device on one of their wrists for seven consecutive nights (concomitantly with sleep diaries), and the mean scores are reported in analyses. Data were scored using the Philips Actiware software version 6.0.9. Philips software algorithms were used to score the data. Data were scored using a 15-second epoch with default settings provided by the manufacturer (10 mins of inactivity for onset of sleep and an awake threshold of 40 counts (medium)) to obtain standard measures of sleep continuity (total sleep time (TST); time in bed (TIB); sleep efficiency (SE%), sleep onset latency (SOL); number of awakenings (NWAK); and wake after sleep onset (WASO)). None of our participants reported extreme (±6 h) disparities between the data provided by the actigraph and sleep diary or provided less than 70% data. One participant did not provide actiwatch data (although this participant provided data for sleep diaries and questionnaires, which are included in the full analyses presented in this paper). The actigraphy data from one further participant revealed an average sleep duration of less than 3 hours (whereas the average of sleep duration based on their sleep diary data was greater than 7 hours). This could reflect a malfunction/misuse of the actiwatch device. Therefore, the actiwatch data for this participant were also excluded from all analyses (of note, as with the other participant who did not provide actiwatch data, the sleep diary and questionnaire data provided by this participant were included in the full analyses presented in this paper).

#### 2.2.3. Pittsburgh Sleep Quality Index (PSQI)

The PSQI is a widely used questionnaire to assess self-reported sleep quality during the previous month [19]. The PSQI comprises seven subscales: (1) subjective sleep quality; (2) sleep latency; (3) sleep duration; (4) habitual sleep efficiency; (5) sleep disturbances; (6) use of sleeping medication; and (7) daytime dysfunction. Scores on these seven subscales range from 0 to 3 and are used to build a global score (ranging from 0 to 21) where higher scores represent poorer self-reported sleep quality [19]. Subjects with a score higher than 5 points can be classified as having poor sleep quality. This questionnaire shows good psychometrics and high correlations with objective measures of sleep [27, 28]. Thirty-eight participants provided data for this questionnaire.

#### 2.2.4. Children's Sleep Habit Questionnaire (CSHQ)

Parents were also asked to report on different aspects of their children's sleep. For this purpose, the CSHQ was used. This questionnaire inquires about child's sleep using 45 items (with three response options: 1—usually; 2—sometimes; and 3—never/rarely) divided in 8 subscales (bed resistance, sleep onset delay, sleep duration, sleep anxiety, night waking, parasomnias, sleep disordered breathing, and daytime sleepiness). Scores on this measure were combined to build a global score (for this global score, 33 items are used) where higher scores indicate more disturbed sleep [29]. Thirty-two participants provided data for this questionnaire.

### 2.3. Statistical Analysis

All the analyses were carried out in R Core Team [30]. Descriptive analyses were performed for sleep diary, actiwatch, and questionnaire data. In order to test for significant differences between CL and SAP, a series of tests were carried out. First, the analyses were restricted to caregiver 1 (*N* = 21) since the main responsibility of T1D management falls on them (these results are presented in the main body of the paper). In order to test differences among CL and SAP in primary caregivers, *T*-test for unpaired samples was used. Second, in the supplementary files, we present ANOVA tests using one factor within subjects (caregiver 1/2) and one factor between subjects (CL or SAP). Results from caregiver 2 are also presented in the supplementary files (Supplementary Tables 1 and 2 and Supplementary Figures 1–3) as well as sensitivity analyses where outliers were excluded. Outliers were identified as a score ±1.5 times the interquartile range (see Supplementary Tables 1 and 2). Because this is not a fully powered study, the discussion of results focuses on the direction of results and effect sizes rather than statistical significance. Of note, none of the analyses reached significance at *p* < 0.05 (see Table 2).

## 3. Results

### 3.1. Sleep Diaries

**Figure 1:**
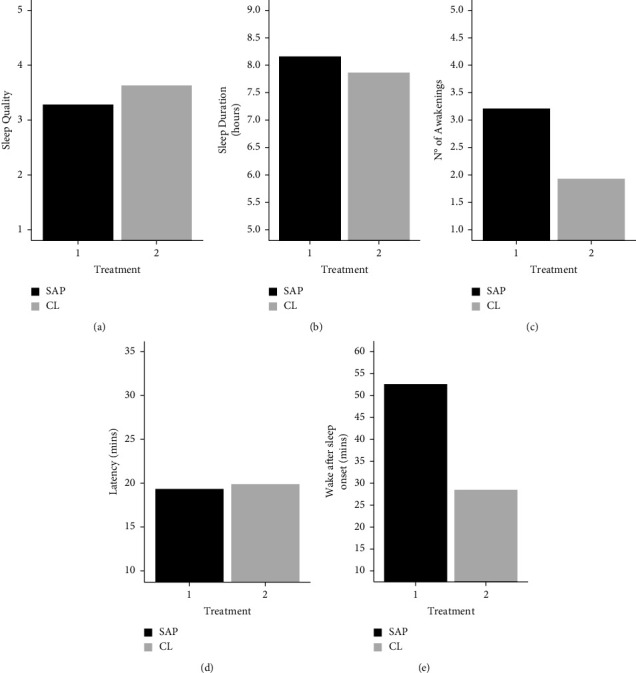
Sleep diary data from primary caregiver by treatment. Treatment refers to whether the children were using a closed-loop system (CL) or sensor-augmented pump (SAP).

Primary caregivers (caregiver 1) from the CL group reported better sleep quality ($\bar{x}$ = 3.6 (SD: 0.4)) as compared to the SAP group ($\bar{x}$ = 3.3 (SD: 0.9)). They also reported fewer awakenings ($\bar{x}$ = 1.9 (SD: 1.1) vs. $\bar{x}$ =  3.2 (SD: 2.5)) and less time awake at night ($\bar{x}$ = 28.6 (SD: 30.9) vs. $\bar{x}$ =  52.6 mins (SD: 48.9)). Primary caregivers from both groups showed a similar SOL ($\bar{x}$ = 19.9 (SD: 18.2) vs. $\bar{x}$ =  19.3 mins (SD: 10.4)), and those from the CL (as compared to the SAP) group reported a shorter sleep duration ($\bar{x}$ = 7.9 (SD: 1.1) vs. $\bar{x}$ =  8.2 hours (SD: 0.7)) (Table 2; Figure 1). Our result also showed that 3 primary carers (15%) had a sleep duration <7 hours (CL = 3), 3 (15%) had a sleep latency >30 mins (SAP = 2, CL = 1), and all carers except one reported an average of more than one awakening per night.

*T*-tests were non-significant in all conditions. However, we found small to moderate effect sizes for all the variables (Cohen's *d* ranging from 0.31 to 0.64)—with the smallest effect size for sleep latency (*d* = 0.04) (Table 2).

#### 3.1.1. Actiwatch

**Figure 2:**
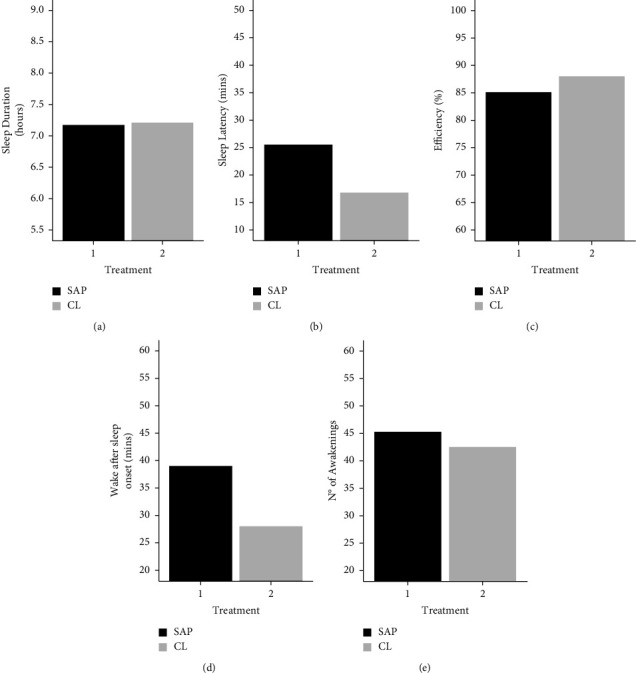
Actiwatch data from primary caregiver by treatment. Treatment refers to whether the children were using a closed-loop system (CL) or sensor-augmented pump (SAP).

Primary caregivers from the CL (as compared to the SAP) group revealed shorter SOL ($\bar{x}$ = 16.7 (SD: 8.6) vs. $\bar{x}$ =  25.5 mins (SD: 11.6)), higher sleep efficiency ($\bar{x}$ = 88.1 (SD: 4.1) vs. $\bar{x}$ =  85.1% (SD: 4.3)) and less WASO ($\bar{x}$ = 28.2 (SD: 11.5) vs. $\bar{x}$ = 39.1 mins (SD: 15.2)). Both groups showed similar sleep duration ($\bar{x}$ = 7.2 (SD: 1.1) vs. $\bar{x}$ =  7.2 hours (SD: 0.9)) and the CL group had fewer awakenings than the SAP group ($\bar{x}$ = 42.5 (SD: 16.7) vs. $\bar{x}$ =  45.3 (SD: 12.2)) (Table 2; Figure 2). Our results showed that 7 carers (35%) had a sleep duration <7 hours (SAP = 3, CL = 4), 5 (25%) had a sleep latency >30 mins (SAP = 4, CL = 1), and 7 (35%) had a sleep efficiency <85% (SAP = 5, CL = 2).

Again, all *T*-tests were non-significant, but moderate to large effect sizes were found for sleep latency (*d* = 0.83), efficiency (*d* = 0.69), and WASO (*d* = 0.78) (Table 2).

#### 3.1.2. Questionnaires

**Figure 3:**
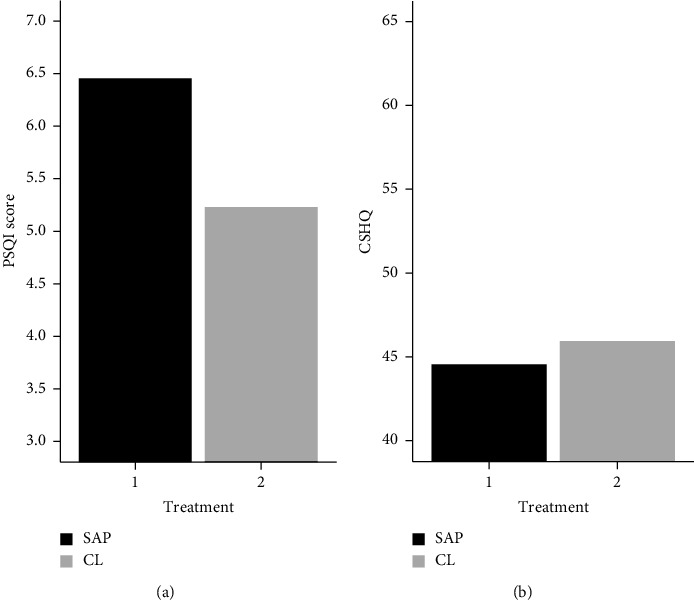
Questionnaire data from primary caregiver by treatment. Treatment refers to whether the children were using a closed-loop system (CL) or sensor-augmented pump (SAP). Sleep quality was measured using the Pittsburgh Sleep Quality Index. CSHQ: Children's Sleep Habit Questionnaire.

Primary caregivers from the CL (as compared to the SAP) group reported better sleep quality (higher scores represent poorer sleep quality) ($\bar{x}$ = 5.2 (SD: 3.3) vs. $\bar{x}$ =  6.5 (SD: 4.1)). Ten carers of those with available data for PSQI (50%) had a PSQI score >5 which is indicative of poor sleep quality (SAP = 7, CL = 3). When primary caregivers reported on their child's sleep (CSHQ), similar values were found in both groups ($\bar{x}$ = 46.0 (SD: 7.2) vs. $\bar{x}$ =  44.5 (SD: 5.4) for CL and SAP groups, respectively) (Table 2; Figure 3). *T*-tests showed no significant differences among the groups. However, as for the other measures, we found small to moderate effect sizes for sleep quality (*d* = 0.31) and the CSHQ scores (*d* = 0.23) (Table 2). Full results including both carers (and sensitivity analyses removing outliers) are presented in Supplementary Tables 1 and 2 and Supplementary Figures 1–3. Additionally, multiple regression models (for all the variables: sleep dairy, actiwatch, and questionnaires) including child age and sex were fitted for the primary caregivers in order to control for their possible effect on the sleep variables, and a similar pattern of non-significant results was found (*p* > 0.05; not reported).

## 4. Discussion

We examined the sleep quality and quantity of a small sample of parents of very young children living with T1D, who were using either a closed-loop system or sensor-augmented pump therapy. We also asked parents to report on their children's overall sleep quality. Given the small sample size, we focused on the general direction of the results and effect sizes (rather than significance), although caution must be taken when using this approach and definitive conclusions should not be drawn. Our results match well with previous research available where a moderate effect for sleep quality improvement was found after the treatment implementation [23, 24] which could reflect a tighter control of child nocturnal glycaemia using closed-loop systems.

Focusing on data from the primary caregiver, there was a general tendency across sleep measures for parents of children using the closed-loop system to report and obtain superior quality sleep to those using the SAP. Some of these differences were of moderate to large effect sizes (>0.6, e.g., SOL, efficiency, or WASO). Such differences, while not significant, could be clinically meaningful, although the limited sample size does not allow us to extract solid conclusions and these relationships could be spurious. Should the finding that the primary caregivers of children using the closed-loop system sleep better than those using the sensor-augmented pump be replicated in a larger sample, this suggests that the benefits of the closed-loop system stem beyond the impact on blood glucose levels and can improve the well-being of the family via other mechanisms (i.e., sleep). This is noteworthy because not only is good sleep quality important for multiple areas of health and well-being but also when caregivers sleep well and avoid sleep deprivation, they are better placed to manage their child's glucose levels [14].

The mechanisms by which the closed-loop system could lead to improved sleep in the primary caregivers were not examined in the context of this study. However, they could include greater glycaemic stability (meaning a reduced need for parental night-time interventions and fewer alarms disturbing sleep during the night) and a reduction in parental anxiety as a result of the reassurance provided by the knowledge that the system tackles both hypo- and hyperglycaemia. This hypothesis is corroborated by results from the main study, which showed that a greater percentage of glucose levels were in the healthy range during night time than during daytime with the closed-loop system [17]. Additionally, a larger proportion of insulin was given automatically by the closed-loop system overnight, compared to more insulin being given manually as a bolus by parents overnight during sensor-augmented pump therapy [17]. Future studies should test empirically possible mechanisms when examining the impact of technology use on sleep in those with T1D and their family members.

The results reported here also underscore the need to consider sleep in the family context [31]. Researchers should consider multiple family members rather than focusing exclusively on the child living with T1D and the primary caregiver—as the impact of using a closed-loop system on sleep quality and quantity could differ depending on the caregiving role within the family (of note, when focusing on caregiver 2 in the supplementary analyses, we did not find as much support that sleep quality appeared to be better in the CL groups as compared to the SAP group). Future work could go further and additionally consider sibling sleep. This too could be impacted by the management of T1D within the household. Conversely, it is also possible that sibling sleep (for example, where there is a new baby in the home) could impact the sleep of the child living with T1D.

The results of this study should be considered in the context of its strengths and limitations. Strengths of this study include the importance of the topic being studied. Those living with T1D and their family members often report short sleep of poor quality [3–5, 7]—and given the significance of sleep for multiple aspects of functioning [32–35], this topic requires urgent research attention. Furthermore, this report focused on parents of young children—spanning key developmental stages, when caregivers are still centrally involved in managing diabetes [24]. Another strength of this study was the focus on sleep 12–14 weeks after the initiation of the treatment being studied. Research suggests that sleep can be particularly impacted soon after a change in diabetes management [4]—meaning that our measurements of sleep did not simply reflect the consequences of a change in management (which is not necessarily applicable to other time periods for which equipment use has become more routine). Finally, the use of multiple methods to measure sleep quality and quantity was an advantage, as a subjective sense of sleep quality can differ from objective measures such as the use of actigraphy [36]. Nonetheless, further work might want to additionally consider other measures such as polysomnography, which would provide another unique perspective on different aspects of sleep in families of those with type 1 diabetes. For example, the proportion of time spent in different sleep stages may differ between families of those using different methods to treat diabetes. Indeed, some parents of children living with T1D have reported that they “sleep lightly” [4].

Despite the many strengths of this study, limitations must also be considered. Perhaps the most significant limitation was the small sample size, which provided limited power to detect group differences for sleep variables. Furthermore, the sleep study was added after the main study had commenced, meaning that it was not possible to use a crossover design. As only a subset of participants was included in this study, those in the two groups were not matched, meaning that factors such as the age of the children in the study could have influenced group differences. Finally, it was noteworthy that most parents in both the SAP and CL groups reported sleep length within recommended guidelines (data from actigraphy also supported this). This might suggest that SAP treatment may itself have resulted in improved T1D management (as compared to multiple daily insulin injections) and supported sleep via a reduction in anxiety for example. This could reduce our ability to detect group differences. Future work might want to additionally also consider sleep quality and quantity in those using SAP and closed-loop systems as well as other modes of insulin delivery.

In summary, while we do not report significant results from this small study, an examination of the direction of the results and effect sizes suggests that the sleep quality of the primary caregiver of children using a closed-loop system was better as compared to that of those using a sensor-augmented pump. Further research needs to further examine this issue in a larger sample before strong conclusions can be drawn. We spend a third of our lives asleep, and sleep taps into different aspects of our health, well-being, and daytime functioning. It is essential that sleep must always be considered when developing technologies to improve the quality of life in people living with T1D and their family members in order to ensure that lives are improved in completeness.

## Figures and Tables

**Table 1:** Children's descriptive statistics.

	SAP	CL	Total
Mean age (SD)	4.1 (1.8)	5.0 (1.6)	4.5 (1.7)
*N* (%), male	8 (80)	6 (54.5)	14 (66.7)
Mean duration of diagnosis in years (SD)	2.4 (1.9)	2.1 (1.5)	2.2 (1.7)
*N* (%), white	8 (80%)	11 (100%)	19 (90)

SD: standard deviation; SAP: sensor-augmented pump; CL: closed-loop system. Two participants endorsed black as their ethnicity.

**Table 2:** *T*-tests comparing closed-loop and sensor-augmented pump groups when results are restricted to caregiver 1.

	Mean SAP	Mean CL	*t*	*p*	Cohen's d
*Sleep diaries*					
Sleep quality	3.3	3.6	−1.147	0.27	0.47
Sleep duration	8.2	7.9	0.714	0.49	0.31
Awakenings	3.2	1.9	1.572	0.14	0.64
Latency	19.3	19.9	−0.085	0.93	0.04
Time awake at night	52.6	28.6	1.265	0.23	0.57

*Actiwatch*					
Sleep duration	7.2	7.2	−0.083	0.93	0.04
Latency	25.5	16.7	1.939	0.07	0.83
Efficiency	85.1	88.1	−1.620	0.12	0.69
WASO	39.1	28.2	1.814	0.09	0.78
Number of awakenings	45.3	42.5	0.417	0.68	0.18

PSQI	6.5	5.2	0.743	0.47	0.31
CSHQ	44.5	46.0	−0.490	0.63	0.23

CSHQ: Children's Sleep Habit Questionnaire; PSQI: Pittsburgh Sleep Quality Index; SAP: sensor-augmented pump; CL: closed-loop system. Number of participants: 21. Sleep quality was reported using a five-point scale from very poor (1) to very good (5), sleep latency, time awake at night, and WASO were coded in minutes, sleep duration was coded in hours, and sleep efficiency was coded in %. Higher scores for PSQI and CSHQ represent poorer sleep quality.

## Data Availability

The data used to support the findings of this study are available from the corresponding author upon reasonable request.

## Supplementary Materials

Supplementary Materials Supplementary Table 1: means (SD) of the sleep variables by treatment group and caregiver order for the sample with and without outliers excluded. Supplementary Table 2: ANOVA tests examining sleep variables by treatment group and caregiver order for the sample with and without outliers. Supplementary Figure 1: sleep diary data from the whole sample by caregiver and treatment. Treatment refers to whether the children were using a closed-loop system (CL) or sensor-augmented pump (SAP), and caregiver refers to whether the parents were considered the primary or secondary caregiver at night. Supplementary Figure 2: actiwatch data from the whole sample by caregiver and treatment. Treatment refers to whether the children were using a closed-loop system (CL) or sensor-augmented pump (SAP), and caregiver refers to whether the parents were considered the primary or secondary caregiver at night. Supplementary Figure 3: questionnaire data from the whole sample by caregiver and treatment. Treatment refers to whether the children were using a closed-loop system (CL) or sensor-augmented pump (SAP), and caregiver refers to whether the parents were considered the primary or secondary caregiver at night. Sleep quality was measured using the Pittsburgh Sleep Quality Index. CSHQ: Children's Sleep Habit Questionnaire.
