# Supplementary material for: Sleep Quality and Quantity in Caregivers of Children with Type 1 Diabetes Using Closed-Loop Insulin Delivery or a Sensor-Augmented Pump
Source: Pediatr Diabetes. 2023 Jun 13;2023:7937007. doi: 10.1155/2023/7937007 (PMC12016902; doi:10.1155/2023/7937007)
Supplement: Supplementary Materials — Supplementary Table 1: means (SD) of the sleep variables by treatment group and caregiver order for the sample with and without outliers excluded. Supplementary Table 2: ANOVA tests examining sleep variables by treatment group and caregiver order for the sample with and without outliers. Supplementary Figure 1: sleep diary data from the whole sample by caregiver and treatment. Treatment refers to whether the children were using a closed-loop system (CL) or sensor-augmented pump (SAP), and caregiver refers to whether the parents were considered the primary or secondary caregiver at night. Supplementary Figure 2: actiwatch data from the whole sample by caregiver and treatment. Treatment refers to whether the children were using a closed-loop system (CL) or sensor-augmented pump (SAP), and caregiver refers to whether the parents were considered the primary or secondary caregiver at night. Supplementary Figure 3: questionnaire data from the whole sample by caregiver and treatment. Treatment refers to whether the children were using a closed-loop system (CL) or sensor-augmented pump (SAP), and caregiver refers to whether the parents were considered the primary or secondary caregiver at night. Sleep quality was measured using the Pittsburgh Sleep Quality Index. CSHQ: Children's Sleep Habit Questionnaire. [file 7937007.f1.zip › 7937007.f1/Supplementary text and Tables_R3.docx]

**Supplementary results**

*Sleep diaries*

Caregiver 2

Parents classified as caregiver 2 showed very similar levels of sleep quality (=3.5 [SD: 0.7] vs =3.5 [SD: 0.9]), sleep duration (=7.7 [SD:0.5] vs =7.5 hours [SD: 1.0]) and SOL (=28.3 [SD: 22.2] vs =27.2 mins [SD: 28.5]) in both groups. However, parents from the CL group reported more awakenings (=2.2 [SD: 1.2] vs =1.6 [SD: 1.3]) and more time awake at night (=27.6 [SD: 34.7] vs =19.1 mins [SD: 16.9]) (Supplementary Table 1; Supplementary Figure 1).

Overall, ANOVA tests showed that the factors (caregiver and treatment) were non-significant for all the sleep diary variables except for awakenings (but only for the analyses with outliers removed) where treatment had a significant impact (p=0.031), the interaction between treatment and caregiver was also significant using the whole sample (p=0.026) but this association did not survive multiple test adjustment (0.05/5= 0.01) (Supplementary Table 2).

*Actiwatch*

Caregiver 2

Focusing on secondary caregivers, they showed shorter SOL in the CL group as compared to the SAP groups (=21.6 [SD: 15.0] vs =39.4 mins [SD: 43.1]). They reported similar sleep duration (=6.7 [SD: 1.2] vs =6.7 hours [SD: 1.2]), sleep efficiency (=82.3 [SD: 9.8] vs =81.8% [SD: 12.7]) and NWAK (=51.2 [SD: 18.5] vs =46.0 [SD: 32.5]). However, parents from the CL group showed more WASO (=49.0 [SD: 27.3] vs =36.1 mins [SD: 41.4]) (Supplementary Table 1; Supplementary Figure 2).

ANOVAs showed that caregiver was a significant factor for sleep duration (p=0.023) and the interaction between treatment and caregiver for WASO (p<0.001) and NWAK (p=0.020). Just the interaction for WASO remained significant after controlling for multiple testing (.05/5=.01) (Supplementary Table 2).

*Questionnaires*

Caregiver 2

Regarding secondary caregivers, sleep quality was similar for the CL group (=6.4 [SD: 4.5]) and the SAP group (=6.3 [SD: 5.3]) and also CSHQ scores (=48.2 [SD: 4.5] vs =47.4 [SD: 5.4]) (Supplementary Table 1; Supplementary Figure 3). The ANOVA test showed no significant differences (Supplementary Table 2).

Supplementary Table 1: Means (SD) of the sleep variables by treatment group and caregiver order for the sample with and without outliers excluded

|  | Treatment | Caregiver | Total sample | Outliers removed |  | Treatment | Caregiver | Total sample | Outliers removed |
| --- | --- | --- | --- | --- | --- | --- | --- | --- | --- |
| **Sleep diaries (Nº outliers)** |  |  |  |  | **Actiwatch (Nº outliers)** |  |  |  |  |
| Sleep quality |  |  |  |  | Sleep duration (4) |  |  |  |  |
|  | SAP | 1 | 3.3 (0.9) |  |  | SAP | 1 | 7.2 (0.9) | 7.4 (0.4) |
|  | CL | 1 | 3.6 (0.4) |  |  | CL | 1 | 7.2 (1.1) | 6.9 (0.7) |
|  | SAP | 2 | 3.5 (0.9) |  |  | SAP | 2 | 6.7 (1.2) | 7.00 (0.9) |
|  | CL | 2 | 3.5 (0.7) |  |  | CL | 2 | 6.7 (1.2) | 7.0 (0.9) |
| Sleep duration (1) |  |  |  |  | Latency (1) |  |  |  |  |
|  | SAP | 1 | 8.2 (0.7) | 8.2 (0.7) |  | SAP | 1 | 25.5 (11.6) | 25.5 (11.6) |
|  | CL | 1 | 7.9 (1.1) | 7.6 (0.9) |  | CL | 1 | 16.7 (8.6) | 16.7 (8.6) |
|  | SAP | 2 | 7.5 (1.0) | 7.5 (1.0) |  | SAP | 2 | 39.4 (43.1) | 26.5 (20.1) |
|  | CL | 2 | 7.7 (0.5) | 7.7 (0.5) |  | CL | 2 | 21.6 (15.0) | 21.6 (15.0) |
| Awakenings (5) |  |  |  |  | Efficiency (3) |  |  |  |  |
|  | SAP | 1 | 3.2 (2.5) | 2.5 (0.6) |  | SAP | 1 | 85.1 (4.3) | 85.1 (4.3) |
|  | CL | 1 | 1.9 (1.1) | 1.7 (0.8) |  | CL | 1 | 88.1 (4.1) | 88.1 (4.1) |
|  | SAP | 2 | 1.6 (1.3) | 2.0 (1.1) |  | SAP | 2 | 81.8 (12.7) | 87.6 (5.5) |
|  | CL | 2 | 2.2 (1.2) | 1.9 (0.6) |  | CL | 2 | 82.3 (9.8) | 85.0 (6.1) |
| Latency (3) |  |  |  |  | WASO (2) |  |  |  |  |
|  | SAP | 1 | 19.3 (10.4) | 19.3 (10.4) |  | SAP | 1 | 39.1 (15.2) | 39.1 (15.2) |
|  | CL | 1 | 19.9 (18.2) | 14.5 (6.5) |  | CL | 1 | 28.2 (11.5) | 28.2 (11.5) |
|  | SAP | 2 | 27.2 (28.5) | 18.0 (7.9) |  | SAP | 2 | 36.1 (41.4) | 22.8 (12.6) |
|  | CL | 2 | 28.3 (22.2) | 22.5 (13.1) |  | CL | 2 | 49.0 (27.3) | 41.8 (17.8) |
| Time awake at night (5) |  |  |  |  | Number of awakenings (2) |  |  |  |  |
|  | SAP | 1 | 52.6 (48.9) | 21.2 (15.7) |  | SAP | 1 | 45.3 (12.2) | 45.3 (12.2) |
|  | CL | 1 | 28.6 (30.9) | 20.3 (17.6) |  | CL | 1 | 42.5 (16.7) | 42.5 (16.7) |
|  | SAP | 2 | 19.1 (16.9) | 19.1 (16.9) |  | SAP | 2 | 46.0 (32.5) | 36.4 (15.8) |
|  | CL | 2 | 27.6 (34.7) | 17.3 (13.0) |  | CL | 2 | 51.2 (18.5) | 46.0 (10.6) |
| **PSQI** |  |  |  |  | **CSHQ** |  |  |  |  |
|  | SAP | 1 | 6.5 (4.1) |  |  | SAP | 1 | 44.5 (5.4) |  |
|  | CL | 1 | 5.2 (3.3) |  |  | CL | 1 | 46.0 (7.2) |  |
|  | SAP | 2 | 6.3 (5.3) |  |  | SAP | 2 | 47.4 (5.4) |  |
|  | CL | 2 | 6.4 (4.5) |  |  | CL | 2 | 48.2 (4.5) |  |

Footnote. SAP: Sensor-augmented pump; CL: Closed-loop. Number of participants: 21 participants classified as caregiver 1 (SAP=11 and CL=10) and 19 participants classified as caregiver 2 (SAP=9 and CL=10). Treatment refers to whether the children were using a closed-loop system or sensor-augmented pump and caregiver refers to whether the parents were considered the primary or secondary caregiver at night. Outliers were identified as a score ±1.5 times the interquartile range. No outliers were identified for the PSQI or the CSHQ (total sample).

Sleep quality was reported using a five-point scale from very poor [1] to very good [5]), sleep latency, time awake at night and WASO were coded in minutes, sleep duration was coded in hours and sleep efficiency in %.

Supplementary Table 2: ANOVA tests examining sleep variables by treatment group and caregiver order for the sample with and without outliers

|  | Total sample | | | | Outliers removed | | |
| --- | --- | --- | --- | --- | --- | --- | --- |
|  | F | P | Eta^2^ | F | | P | Eta^2^ |
| **Sleep diaries (Nº outliers)** |  |  |  |  | |  |  |
| Sleep Quality (0) |  |  |  |  | |  |  |
| Treatment | 0.568 | 0.461 | 0.024 |  | |  |  |
| Caregiver | 0.112 | 0.742 | 0.002 |  | |  |  |
| Treatment* Caregiver | 1.847 | 0.192 | 0.029 |  | |  |  |
| Sleep duration (1) |  |  |  |  | |  |  |
| Treatment | 0.002 | 0.962 | <0.001 | 0.203 | | 0.658 | 0.008 |
| Caregiver | 2.046 | 0.171 | 0.038 | 1.241 | | 0.282 | 0.026 |
| Treatment* Caregiver | 0.783 | 0.388 | 0.015 | 1.892 | | 0.188 | 0.039 |
| Awakenings (5) |  |  |  |  | |  |  |
| Treatment | <0.001 | 0.997 | <0.001 | **5.720** | | **0.031*** | **0.153** |
| Caregiver | 1.670 | 0.214 | 0.025 | 0.849 | | 0.372 | 0.033 |
| Treatment* Caregiver | **5.940** | **0.026*** | **0.083** | 2.664 | | 0.125 | 0.096 |
| Latency (3) |  |  |  |  | |  |  |
| Treatment | 0.002 | 0.968 | <0.001 | 0.096 | | 0.761 | 0.005 |
| Caregiver | 2.051 | 0.170 | 0.036 | 1.265 | | 0.280 | 0.030 |
| Treatment* Caregiver | 0.023 | 0.882 | <0.001 | 1.393 | | 0.258 | 0.033 |
| Time awake at night (5) |  |  |  |  | |  |  |
| Treatment | 0.038 | 0.847 | 0.002 | 0.028 | | 0.870 | 0.001 |
| Caregiver | 3.203 | 0.092 | 0.046 | 0.650 | | 0.435 | 0.024 |
| Treatment* Caregiver | 2.765 | 0.116 | 0.040 | 0.097 | | 0.761 | 0.004 |
| **Actiwatch (Nº outliers)** |  |  |  |  | |  |  |
| Sleep duration (4) |  |  |  |  | |  |  |
| Treatment | 0.091 | 0.768 | 0.004 | 0.047 | | 0.832 | 0.002 |
| Caregiver | **6.366** | **0.023*** | **0.131** | 2.241 | | 0.160 | 0.068 |
| Treatment* Caregiver | 0.109 | 0.745 | 0.003 | 0.304 | | 0.591 | 0.010 |
| Latency (1) |  |  |  |  | |  |  |
| Treatment | 3.375 | 0.086 | 0.117 | 2.237 | | 0.157 | 0.099 |
| Caregiver | 1.197 | 0.182 | 0.051 | 0.881 | | 0.364 | 0.019 |
| Treatment* Caregiver | 0.320 | 0.580 | 0.009 | 0.411 | | 0.532 | 0.009 |
| Efficiency (3) |  |  |  |  | |  |  |
| Treatment | 0.627 | 0.441 | 0.026 | 0.020 | | 0.890 | 0.001 |
| Caregiver | 3.444 | 0.083 | 0.075 | 0.198 | | 0.664 | 0.005 |
| Treatment* Carer | 0.367 | 0.554 | 0.009 | 3.984 | | 0.069 | 0.088 |
| WASO (2) |  |  |  |  | |  |  |
| Treatment | <0.001 | 0.991 | <0.001 | 0.942 | | 0.349 | 0.044 |
| Caregiver | 0.568 | 0.463 | 0.014 | 0.782 | | 0.392 | 0.022 |
| Treatment* Caregiver | 3.444 | 0.087 | 0.076 | **18.581** | | **<0.001**** | **0.346** |
| Number of Awakenings (2) |  |  |  |  | |  |  |
| Treatment | 0.036 | 0.852 | 0.001 | 0.580 | | 0.460 | 0.030 |
| Carer | 0.078 | 0.784 | 0.002 | 2.014 | | 0.179 | 0.047 |
| Treatment* Caregiver | 1.041 | 0.324 | 0.026 | **7.016** | | **0.020*** | **0.146** |
| **PSQI** |  |  |  |  | |  |  |
| Treatment | 0.068 | 0.798 | 0.004 |  | |  |  |
| Caregiver | 0.000 | 1 | 0.000 |  | |  |  |
| Treatment* Caregiver | 0.427 | 0.524 | 0.017 |  | |  |  |
| **CSHQ** |  |  |  |  | |  |  |
| Treatment | 0.758 | 0.406 | 0.070 |  | |  |  |
| Caregiver | 0.045 | 0.837 | 0.001 |  | |  |  |
| Treatment* Caregiver | 0.193 | 0.671 | 0.002 |  | |  |  |

Note. CSHQ: Children’s Sleep Habit Questionnaire; PSQI: Pittsburgh Sleep Quality Index; WASO = wake after sleep onset. Number of participants: 21 participants classified as caregiver 1 (SAP=11 and CL=10) and 19 participants classified as caregiver 2 (SAP=9 and CL=10). Treatment refers to whether the children were using a closed-loop system or sensor-augmented pump and caregiver refers to whether the parents were considered the primary or secondary caregiver at night. Outliers were identified as a score ±1.5 times the interquartile range. No outliers were identified for the PSQI or the CSHQ (total sample).

* significance level p<.05; **significance level p<.001.
