# Supplementary figures and images for: Sleep Quality and Quantity in Caregivers of Children with Type 1 Diabetes Using Closed-Loop Insulin Delivery or a Sensor-Augmented Pump
Source: Pediatr Diabetes. 2023 Jun 13;2023:7937007. doi: 10.1155/2023/7937007 (PMC12016902; doi:10.1155/2023/7937007)

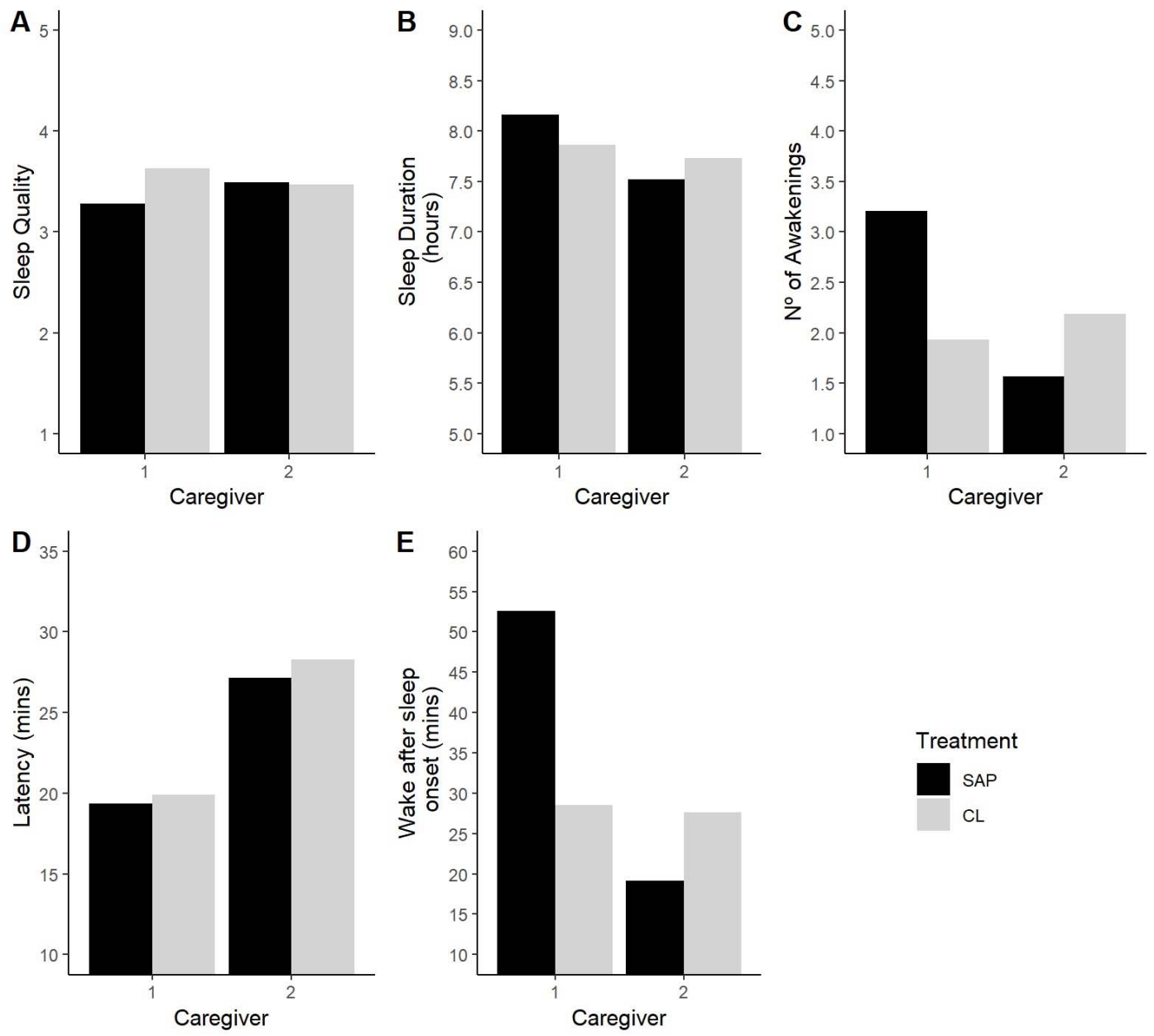

Supplement: Supplementary Materials — Supplementary Table 1: means (SD) of the sleep variables by treatment group and caregiver order for the sample with and without outliers excluded. Supplementary Table 2: ANOVA tests examining sleep variables by treatment group and caregiver order for the sample with and without outliers. Supplementary Figure 1: sleep diary data from the whole sample by caregiver and treatment. Treatment refers to whether the children were using a closed-loop system (CL) or sensor-augmented pump (SAP), and caregiver refers to whether the parents were considered the primary or secondary caregiver at night. Supplementary Figure 2: actiwatch data from the whole sample by caregiver and treatment. Treatment refers to whether the children were using a closed-loop system (CL) or sensor-augmented pump (SAP), and caregiver refers to whether the parents were considered the primary or secondary caregiver at night. Supplementary Figure 3: questionnaire data from the whole sample by caregiver and treatment. Treatment refers to whether the children were using a closed-loop system (CL) or sensor-augmented pump (SAP), and caregiver refers to whether the parents were considered the primary or secondary caregiver at night. Sleep quality was measured using the Pittsburgh Sleep Quality Index. CSHQ: Children's Sleep Habit Questionnaire. [file 7937007.f1.zip › 7937007.f1/Supplementary Figure 1_R1.pdf]

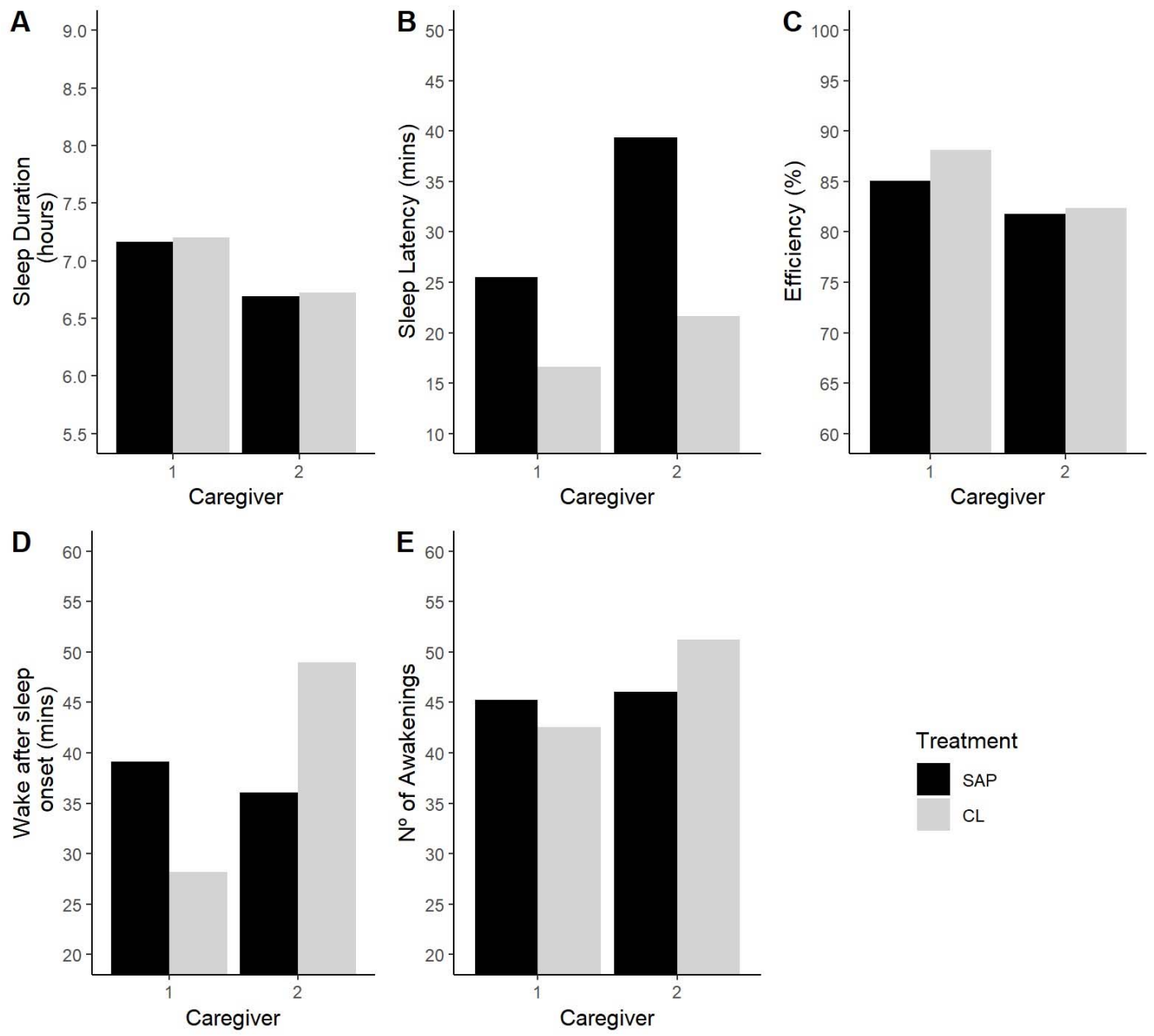

Supplement: Supplementary Materials — Supplementary Table 1: means (SD) of the sleep variables by treatment group and caregiver order for the sample with and without outliers excluded. Supplementary Table 2: ANOVA tests examining sleep variables by treatment group and caregiver order for the sample with and without outliers. Supplementary Figure 1: sleep diary data from the whole sample by caregiver and treatment. Treatment refers to whether the children were using a closed-loop system (CL) or sensor-augmented pump (SAP), and caregiver refers to whether the parents were considered the primary or secondary caregiver at night. Supplementary Figure 2: actiwatch data from the whole sample by caregiver and treatment. Treatment refers to whether the children were using a closed-loop system (CL) or sensor-augmented pump (SAP), and caregiver refers to whether the parents were considered the primary or secondary caregiver at night. Supplementary Figure 3: questionnaire data from the whole sample by caregiver and treatment. Treatment refers to whether the children were using a closed-loop system (CL) or sensor-augmented pump (SAP), and caregiver refers to whether the parents were considered the primary or secondary caregiver at night. Sleep quality was measured using the Pittsburgh Sleep Quality Index. CSHQ: Children's Sleep Habit Questionnaire. [file 7937007.f1.zip › 7937007.f1/Supplementary Figure2_R1.pdf]

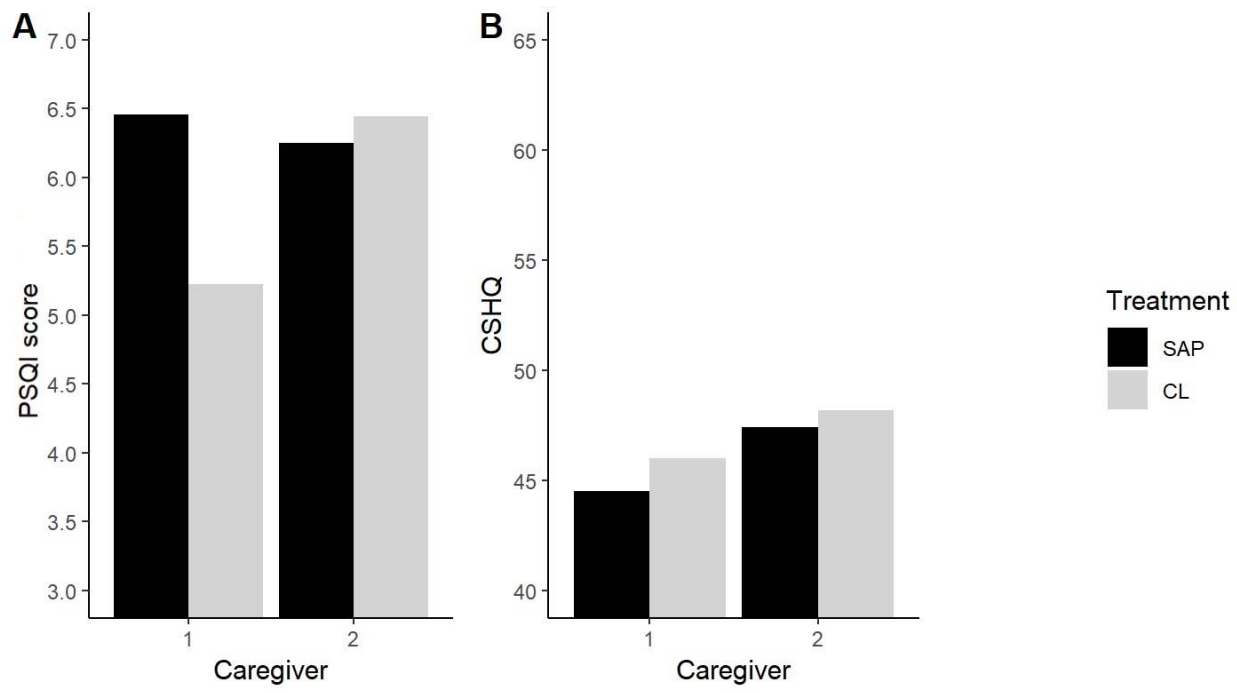

Supplement: Supplementary Materials — Supplementary Table 1: means (SD) of the sleep variables by treatment group and caregiver order for the sample with and without outliers excluded. Supplementary Table 2: ANOVA tests examining sleep variables by treatment group and caregiver order for the sample with and without outliers. Supplementary Figure 1: sleep diary data from the whole sample by caregiver and treatment. Treatment refers to whether the children were using a closed-loop system (CL) or sensor-augmented pump (SAP), and caregiver refers to whether the parents were considered the primary or secondary caregiver at night. Supplementary Figure 2: actiwatch data from the whole sample by caregiver and treatment. Treatment refers to whether the children were using a closed-loop system (CL) or sensor-augmented pump (SAP), and caregiver refers to whether the parents were considered the primary or secondary caregiver at night. Supplementary Figure 3: questionnaire data from the whole sample by caregiver and treatment. Treatment refers to whether the children were using a closed-loop system (CL) or sensor-augmented pump (SAP), and caregiver refers to whether the parents were considered the primary or secondary caregiver at night. Sleep quality was measured using the Pittsburgh Sleep Quality Index. CSHQ: Children's Sleep Habit Questionnaire. [file 7937007.f1.zip › 7937007.f1/Supplementary Figure3_R1.pdf]
